# Expression levels of ROS1/ALK/c-MET and therapeutic efficacy of cetuximab plus chemotherapy in advanced biliary tract cancer

**DOI:** 10.1038/srep25369

**Published:** 2016-05-03

**Authors:** Nai-Jung Chiang, Chiun Hsu, Jen-Shi Chen, Hsiao-Hui Tsou, Ying-Ying Shen, Yee Chao, Ming-Huang Chen, Ta-Sen Yeh, Yan-Shen Shan, Shiu-Feng Huang, Li-Tzong Chen

**Affiliations:** 1 Institute of Clinical Medicine, College of Medicine, National Cheng Kung University, Tainan, Taiwan; 2 National Institute of Cancer Research, National Health Research Institutes, Tainan, Taiwan; 3 Division of Hematology and Oncology, Department of Internal Medicine, National Cheng Kung University Hospital, Tainan, Taiwan; 4 Department of Oncology, National Taiwan University Hospital, and National Taiwan University Cancer Center, Taipei, Taiwan; 5 Graduate Institute of Oncology, National Taiwan University College of Medicine, Taipei, Taiwan; 6 Division of Hematology and Oncology, Department of Internal Medicine, Linkou Chang Gung Memorial Hospital and Chang Gung University, Taoyuan, Taiwan; 7 Division of Biostatistics and Bioinformatics, Institute of Population Health Sciences, National Health Research Institutes, Miaoli, Taiwan; 8 Graduate Institute of Biostatistics, College of Public Health, China Medical University, Taichung, Taiwan; 9 Institute of Molecular and Genomic Medicine, National Health Research Institutes, Miaoli, Taiwan; 10 Department of Oncology, Taipei Veterans General Hospital, Taipei, Taiwan; 11 Department of Surgery, Linkou Chang Gung Memorial Hospital and Chang Gung University, Taoyuan, Taiwan; 12 Department of Surgery, National Cheng Kung University Hospital, Tainan, Taiwan; 13 Institute of Clinical Pharmacology, College of Medicine, National Cheng Kung University, Tainan, Taiwan; 14 Department of Internal Medicine, Kaohsiung Medical University Hospital, Kaohsiung Medical University, Kaohsiung, Taiwan

## Abstract

Aberrant expression of ROS1, ALK or c-MET (RAM) is implicated in carcinogenesis and cancer drug resistance. We retrospectively evaluated the effect of RAM expression on outcomes for advanced biliary tract cancer patients, who were treated with gemcitabine plus oxaliplatin (GEMOX), with or without cetuximab, in a randomized phase II trial. RAM expression levels on archived tissue sections were scored using immunohistochemistry (IHC). Of 110 tumors with IHC staining for all three markers, 18 were RAM^high^ (IHC intensity 3+ for any markers). Ninety-two tumors were RAM^low^ (IHC intensity <3+ for all markers). All RAM^high^ tumors were intra-hepatic cholangiocarcinomas (IHCC). Of the patients with IHCC (n = 80), median overall survival (OS) of RAM^high^ group was inferior to that of the RAM^low^ group (5.7 *vs.* 11.7 months*, p* = 0.021). In multivariate analysis RAM^high^ remained an independently adverse prognostic factor, with a hazard ratio of 2.01 (*p* = 0.039). In the RAM^low^ group, GEMOX treatment with cetuximab significantly improved the disease control rate (68% *vs.* 41%, *p* = 0.044), median progression-free survival (7.3 *vs.* 4.9 months*, p* = 0.026), and marginally prolonged median OS (14.1 vs 9.6 months, *p* = 0.056), compared to GEMOX treatment alone. Future trials of anti-EGFR inhibitors for IHCC may consider RAM expression as a patient stratification factor.

Systemic chemotherapy with gemcitabine plus platinum is recognized as the standard of care for patients with advanced or metastatic biliary tract cancer (ABTC)^1^. However, treatment outcomes are largely unsatisfactory with a median overall survival (OS) ranging from 7 to 11 months^2^,^3^. Combination of inhibitors targeting the epidermal growth factor receptor (EGFR) pathway with chemotherapy has been extensively studied to improve the efficacy of chemotherapy^4^,^5^,^6^,^7^. Although trends of improved objective response rate and progression-free survival (PFS) had been observed in certain trials, none of these including “all comers” randomized trials could demonstrate the survival benefit of add-on anti-EGFR inhibitors in ABTC patients receiving gemcitabine plus platinum chemotherapy^4^,^5^,^7^. Identification of predictive and prognostic biomarkers is crucial to better define patients who may benefit from targeted molecular therapies such as anti-EGFR inhibitors.

Various biomarkers predictive for anti-EGFR inhibitor efficacy have been explored for different cancer types. For example, *RAS*/*BRAF* mutations predicted poor response to cetuximab in patients with metastatic colorectal cancers^8^. In another study, high EGFR expression predicted incremental benefit from cetuximab combine with chemotherapy in patients with advanced non-small cell lung cancer (NSCLC)^9^. However, two previous randomized studies with either pre-planned or post-hoc analyses failed to demonstrate an association between the status of *RAS*/*BRAF* mutations and the therapeutic efficacy of cetuximab in ABTC, or between the latter and EGFR expression^4^,^7^. Additional molecular alternations have been identified to account for the resistance to EGFR inhibition in NSCLC and provide opportunities for new drug development. Among them, over-expression of c-MET, which could result in activation of hepatocyte growth factor (HGF)-c-MET signaling correlates with resistance to anti-EGFR therapy in patients with NSCLC^10^. c-MET overexpression has been detected in 11.7% and 16.2% of intra- and extra-hepatic cholangiocarcinoma (IHCC and EHCC) cases, respectively, and associated with inferior post-resection recurrence-free survival than those without^11^. Chromosome rearrangements involving either anaplastic lymphoma kinase (*ALK*) or *ROS1* genes have been identified in wild-type *EGFR* NSCLCs that were refractory to anti-EGFR therapy^12^,^13^. *ROS1* rearrangement has been detected in 8.7% of IHCC cases and shown to be oncogenic in the BTC mouse model, whereas *ALK* rearrangement or aberrant protein expression has not been described in ABTC^14^,^15^. In NSCLC, *ALK* or *ROS1* rearrangement-harboring tumors exhibited compatible response to ALK inhibitors because of the high concordance of kinase domain sequences^16^,^17^. Whether alternations in c-MET, ROS1, and ALK signaling pathways may impact the clinical outcomes of patients undergoing anti-EGFR therapy and provide opportunities for new ABTC drug development, remains unexplored.

The present *post-hoc* study evaluates predictive and prognostic significances of aberrant ROS1, ALK or c-MET (RAM) expression in a prospective cohort of ABTC patients who received gemcitabine plus oxaliplatin (GEMOX) chemotherapy with or without cetuximab in a randomized phase II study.

## Results

### RAM protein expression and rearrangement of *ROS1* and *ALK* genes

**Figure 1:**
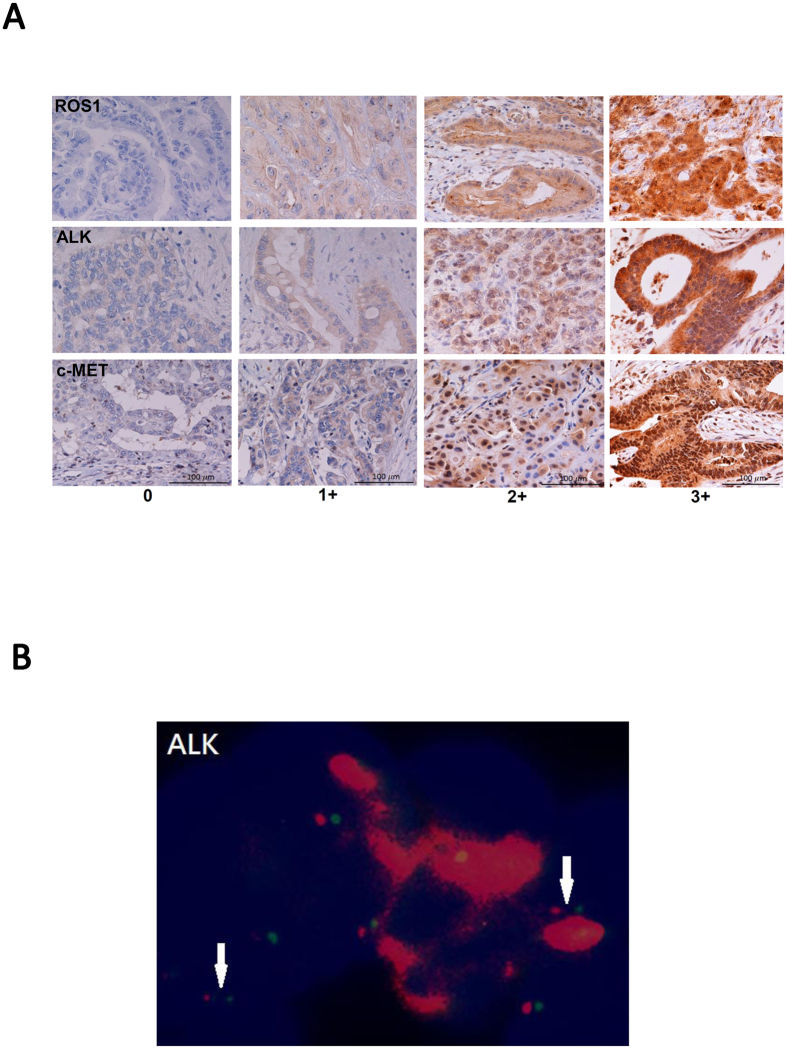
(**A**) ROS1, ALK and c-MET expression in primary intra-hepatic cholangiocarcinoma (expression score: 0, 1+, 2+ and 3+). ROS1 and ALK are mainly localized in cytoplasm, and c-MET is localized in both cytoplasm and nucleus. (**B**) Break-apart Fluorescence *in situ* hybridization for *ALK* revealed wide-apart of the red and green signals (white arrows), indicating *ALK* translocation.

Of the tumor tissue sections from 122 patients who participated in the prospective randomized phase II trial, sections from 110 patients had sufficient tissue for IHC of all three markers. The staining of the RAM markers was mainly localized in the cytoplasm of tumor cells (Fig. 1A). Among them, 18 of cases (16%) were categorized as RAM^high^, with 3+ IHC staining of ROS1 in 9 cases, ALK in 5, and c-MET in 6 (Supplementary Table 1). One tumor overexpressed all three markers. *ALK* rearrangement was detected in 1 of the 5 tumors presenting ALK overexpression (Fig. 1B), whereas none of the 9 tumors with ROS1 overexpression had detectable *ROS1* rearrangement.

### Correlations between RAM expression and clinicopathological factors

As of December 31, 2014, the median follow-up was 10.3 months (range 0.9–45.4 months). The demography of the 110 ABTC patients with RAM^high^ or RAM^low^ tumors is listed in Table 1. Compared with RAM^low^ tumors, RAM^high^ tumors were more likely to be IHCC (100% *vs.* 67.4%, *p* = 0.015), without prior surgery (0% *vs.* 48.9%, *p* < 0.0001), and with concurrent mutant *KRAS* (55.6% *vs.* 31.5%, *p* = 0.063). The trend of differences in the baseline characteristics between RAM^high^ and RAM^low^ groups was similar in the 80 patients with IHCC (Supplementary Table 2).

### Comparison of treatment efficacies

**Figure 2:**
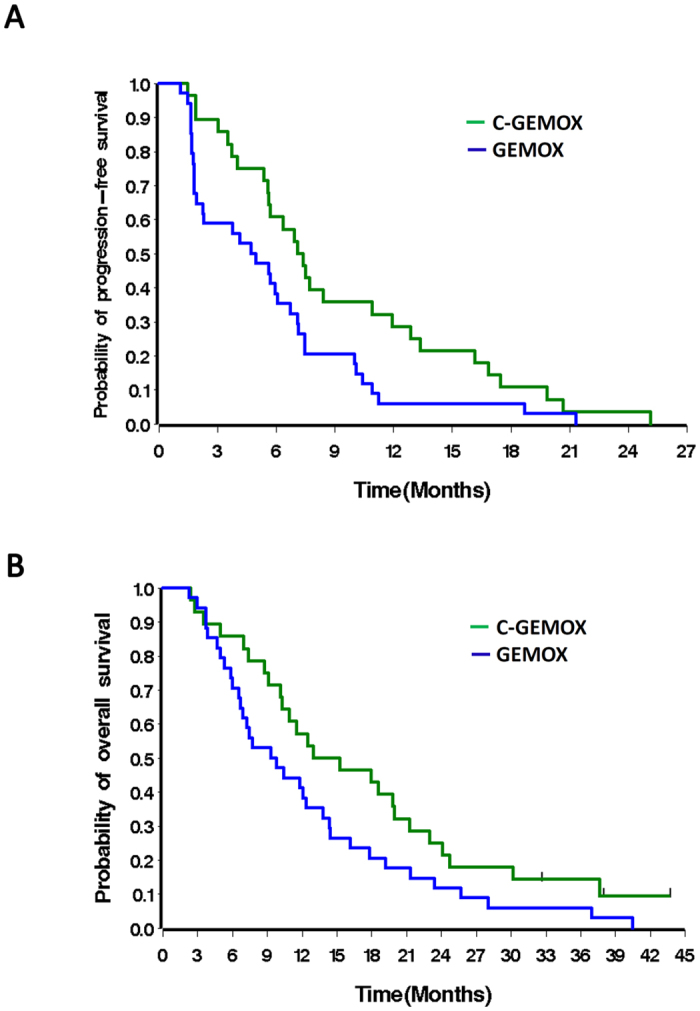
The comparison of PFS (**A**) and OS (**B**) in IHCC patients with RAM^low^ -expressed tumors (n = 62), receiving treatment of C-GEMOX or GEMOX. (**C**) cetuximab; GEMOX, gemcitabine and oxaliplatin; PFS, progression-free survival; OS, overall survival; IHCC, intrahepatic cholangiocarcinoma; RAM, ROS1/ALK/c-MET.

Of the 110 patients included in this study, those who received C-GEMOX had a tended to have better ORR, better long-term disease control rates (DCR^≥16wk^, objective response according to RECIST plus stable disease [SD] ≥ 16 weeks), and longer survival compared to patients who received GEMOX (Supplementary Table 3)^2^. Because all RAM^high^ tumors were IHCCs, further analysis was performed on the IHCC population. Despite similar DCR^≥16wk^ and PFS, IHCC patients with RAM^high^ tumors had a significantly inferior median OS than patients with RAM^low^ tumors (5.7 *vs.* 11.7 months, *p* = 0.021, Table 2). In the RAM^low^ IHCC subgroup, C-GEMOX treatment achieved significantly better DCR^≥16wk^ (68% *vs.* 41%, *p* = 0.044) and median PFS (7.3 *vs.* 4.9 months, *p* = 0.026) than GEMOX alone did. A trend toward improved OS was also observed (14.1 *vs.* 9.6 months, *p* = 0.056, Table 3 and Fig. 2). Both *KRAS*^*wt*^ and *KRAS*^*mut*^ patients in the RAM^low^ IHCC subgroup showed similar trends of improvement in DCR^≥16wk^ and median PFS after C-GEMOX treatment (Supplementary Table 4). This *KRAS*-independent improvement suggests that *KRAS* mutation did not interfere with the benefits derived from add-on cetuximab to GEMOX in RAM^low^ cases. The findings were consistent with our previous report on the entire ABTC intention-to treat population^4^. However, in the RAM^high^ subgroup, the DCR^≥16wk^, PFS and OS were similar between C-GEMOX and GEMOX-treated patients (Table 3).

Multivariate analysis showed that male gender and RAM^high^ subgroup were independent adverse prognostic factors for OS in 80 IHCC patients (Table 4). Male gender and GEMOX alone had only a statistically non-significant tendency to prognosticate worse PFS (Supplementary Table 5).

### Recommendations for phase III trial design based on phase II trial with biomarkers

In the RAM^low^ IHCC subgroup, C-GEMOX achieved better median PFS than GEMOX did, with a hazard ratio (HR) of 1.78 at one-sided α = 0.1, and the null hypothesis was rejected, according to Freidlin *et al.*^17^. The 2-sided 80% confidence interval (CI) for the HR in the RAM^high^ subpopulation was 0.39 to 1.50 (Supplementary Table 6). For RAM^high^ cases, this range is consistent with the null hypothesis that the efficacy of C-GEMOX is no different from that of GEMOX. Therefore, future large-scale clinical trials should consider using RAM expression as a patient stratification factor.

## Discussion

The present *post-hoc* analysis is the first report to describe the prognostic significance of aberrant ROS1, ALK, or c-MET expression in ABTC patients undergoing front-line therapy. We found that patients with tumors over-expressing any of the ROS1, ALK, or c-MET proteins (RAM^high^) had inferior OS, regardless of the assigned treatment. Patients with RAM^low^ tumors, notably of IHCC, were likely to benefit from the addition of cetuximab to GEMOX chemotherapy.

The findings of 18 RAM^high^ tumors that were all IHCC cases and without prior surgery at study entry imply that aberrant RAM expression can be site-specific and associated with aggressive tumor biology. Recent comprehensive genomic profiling studies of BTCs also suggested distinct etiologies and molecular characteristics among IHCC, EHCC and gallbladder carcinoma (GBC)^18^,^19^,^20^,^21^. *IDH* mutation and *FGFR* rearrangement are usually specific to IHCC, whereas *BAP1* and *IDH* mutations are more frequently detected in non-parasite related cholangiocarcinoma than in those related to parasite infection. Compared with IHCC, EHCC has a significantly higher frequency of *TP53* and *KRAS* mutations. These BTC subtype-specific genetic alternations indicate that studies of molecular pathogenesis and therapeutic development may be most fruitful on a subtype-specific basis. The observation of higher *KRAS* mutation rate in RAM^high^ tumors indicates that the RAM^high^ phenotype might be related to the ‘proliferation’ class of IHCC, which is associated with worse clinical outcomes than other IHCC classes^22^,^23^.

Because of the lack of association between the status of *KRAS* mutation or EGFR positivity and therapeutic benefit of cetuximab as shown in our primary report, we strongly encourage a search for other predictive biomarkers for anti-EGFR therapy in ABTC. ROS1, ALK and c-MET, appear to be reasonable choices because the known crosstalk between EGFR signaling and all three signaling pathways, and their genetic alterations or aberrant expression may confer resistance to EGFR inhibition in NSCLC or other cancer types^24^,^25^,^26^,^27^,^28^,^29^. For example, c-MET activation involves the acquisition of resistance to anti-EGFR therapy via bypass of the EGFR pathway^26^. EGFR activation can mediate the resistance to ROS1 inhibition in NSCLC^25^. In addition, ROS1 activation mediates resistance to EGFR inhibition in glioblastoma cells^24^. ALK kinase is another druggable target in NSCLC patients who do not respond to EGFR inhibitors or have *EGFR* wild-type tumors; ALK activation may be associated with resistance to EGFR tyrosine kinase inhibitor^27^,^28^. Although aberrant ALK signaling has not been reported in BTC, *ROS1* rearrangement and c-MET over-expression have been detected in cholangiocarcinoma; the latter observations are associated with aggressive biological behavior^11^,^14^,^15^. For clinically available inhibitors of receptor tyrosine kinase targeting either ALK/ROS1 or ALK/ROS1 and c-MET^30^,^31^, we include ALK expression in our analysis. Finally, due to the low respective incidence of aberrant ROS1, ALK, or c-MET expression, a pooled analysis of all 3 molecules was performed to increase the statistical power of our analysis.

Although our previous studies demonstrated a high concordance between *ALK* rearrangement and protein over-expression in surgically resected NSCLC samples^32^, FISH detected chromosomal translocation for only 1 of 5 ALK over-expression tumors in the present study (*ALK* rearrangement, Fig. 1B). However, our present finding of discord between the frequency of ROS1 over-expression and that of *ROS1* rearrangement is consistent with results of previous IHCC and NSCLC studies. Seventy-two of 194 surgically resected IHCC tumors (37%) exhibited high expression level of ROS1; none of the 102 samples successfully analyzed using FISH had detectable *ROS1* rearrangement^33^. Analyses of NSCLC tumors found that the frequency of ROS1 over-expression and *ROS1* rearrangement was 22% (9 of 399) and 1.7% (18 of 1037), respectively^12^,^34^. Lee *et al.* further showed that a small fraction of NSCLC tumors over-expressing ROS1 exhibited hypo-methylation in the *ROS1* promoter region; however, the mechanisms of this dysregulation remain largely un-elucidated^34^. Our discordant IHC and FISH results could be caused by the technical difficulty posed by FISH analyses of small IHCC biopsy tissue samples. Another possible explanation of the discordance could be that translational or post-translational regulation enhances the expression of ROS1/ALK proteins.

Our data indicate that RAM^low^ expression predicts therapeutic efficacy of add-on cetuximab in IHCC with GEMOX-based therapy. These results are applicable to this statistical model proposed by Freidlin *et al.*, which aims to clarify the values off biomarkers in designing phase III randomized trials of targeted therapies^35^. RAM expression status should be considered as a stratification factor (RAM^low^ *vs.* RAM^high^ subgroups) for future trials of anti-EGFR inhibitors in IHCC. However, the wide 80% CI of the HR estimate (Supplementary Table 6), resulting from the small sample size of patients in the RAM^high^ subgroup, possibly misled the conclusion according to Freidlin’s algorithm. Therefore, we strongly recommend adoption of this algorithm at the interim analysis of phase III studies, through monitoring the HR of biomarker-defined subgroups. Such monitoring should be used to determine whether RAM should be used continuously as a stratification factor or shifted to the role of an enrichment factor. Based on the dismal survival outcome of RAM^high^ patients and the well-known clinical activity of ALK inhibitors in NSCLC with *ALK* and/or *ROS1* rearrangement, an investigator-initiated phase II trial is ongoing. This trial evaluates the efficacy and safety of ceritinib in patients with IHCC over-expressing ROS1 or ALK (clinicaltrials.gov NCT2374489).

For IHCC patients, ROS1, ALK and c-MET expression levels have prognostic significance on clinical outcomes. Although this finding may require further validation, it has led to proposal of a new stratification or enriched biomarker for future phase III trial of anti-EGFR therapy in IHCC. This example demonstrates the importance of prospective tissue sample collection from well-designed clinical trials for companion exploratory biomarker studies. Such exploratory studies can facilitate further drug development.

## Methods

### Patients

The current study analyzed archival tumor tissues from patients with ABTC who participated in a Taiwan Cooperative Oncology Group (TCOG)-sponsored randomized phase II trial comparing first-line GEMOX alone versus GEMOX plus cetuximab (C-GEMOX)^4^. In brief, 122 patients were 1:1 randomized, stratified by tumorous *KRAS* status, ECOG performance status, and location of primary tumor, and then 1:1 randomized. The patients received GEMOX or C-GEMOX until disease progression or the occurrence of unacceptable toxicity. The primary end point of the study was objective response rate (ORR) by RECIST. Trends toward improvement in ORR (27% vs. 15%, *p* = 0.12) and median PFS (6.7 vs. 4.1 months, *p* = 0.05) favoring C-GEMOX were observed. The trends of improvement did not correlate with tumorous *KRAS* mutation status. However, C-GEMOX did not improve OS (10.6 vs. 9.8 months, *p* = 0.91) at primary analysis with a cut-off date on April 30, 2013^4^. In the present study, survival follow-up was updated to December 31, 2014. Tumor procurement was approved by the institutional review board of National Health Research Institutes (EC1040427-E). Written informed consent was obtained from all subjects and the study was conducted in accordance with International Conference Statements for Good Clinical Laboratory Practice and the declaration of Helsinki of the World Health Medical Association.

### *KRAS* mutation analysis

Genomic DNA was extracted from tumor tissues using TaKaRa DEXPAT^TM^ (TaKaRa Bio Inc, Japan). *KRAS* mutation status was then determined using direct sequencing of the coding sequences of exons 2 and 3, as well as the TaqMan assay for *KRAS* codon 12/13 mutations using the LightMix Kit (TIB MOLBIOL GmbH, Germany). Tumors were considered to be *KRAS* mutation if any test was positive.

### Immunohistochemical study

Formalin-fixed, paraffin-embedded (FFPE) tumor tissue sections were subjected to immunohistochemistry (IHC) to determine RAM expression levels. In brief, 4-μm-thick tissue sections were de-paraffinized, followed by antigen retrieval (Cell Conditioning 1, Roche, Rotkreuz, Switzerland) for 10 minutes (for ROS1 staining) or 15 minutes (for ALK and c-MET staining) at 95 °C. The slides were then incubated with primary antibody against ROS1 (Abcam plc., Cambridge, UK, in 1:100 dilution for 1 hour), c-MET (clone C12, Biotechnology, Inc., Santa Cruz, Calif., in 1:200 dilution for 1 hour) or ALK (clone ZAL4, Invitrogen, Life Tech Corp, Carlsbad, Calif., in 1:500 dilution for 16 hours). The detection was performed by the Ventana Discovery XT staining system with ultraView Universal DAB detection kit (Ventana Medical Systems, Inc, Tucson, AZ) according to the manufacturer’s protocol. Two pathologists (YYS and SFH), who were blinded to the clinical information, independently reviewed and scored the IHC staining results. The expression levels of ROS1, ALK and c-MET in tumor tissue was graded as negative (0), weak (1+), moderate (2+), or high (3+), according to the highest staining intensity identified in more than 10% of the tumor tissue. Tumors with high expression level (3+ staining intensity) of any one of the RAM markers were categorized as RAM^high^ tumors; those with 0 to 2+ staining intensity for all 3 markers were categorized as RAM^low^ tumors. EGFR protein expression was determined by immunohistochemical (IHC) staining (primary antibody: DAKO, H11 Cytomation Denmark A/S, Glostrup, Denmark), followed by staining with the ultraView Universal DAB detection kit (Ventana Medical Systems, Inc., Tucson, AZ, USA). EGFR protein expression was considered positive if ≥1% of tumor cells were staining.

### Fluorescence *in situ* hybridization (FISH)

Tumors over-expressing ROS1 or ALK (3+) in IHC were subjected to FISH studies as previously described^32^. The probes for detecting *ALK* and *ROS1* translocation were Vysis ALK Break Apart FISH Probe (Abbott Molecular Inc. Des Plaines, IL, USA) and ROS1 Split FISH probe (Abnova Biotec Co., Ltd, Taiwan), respectively. At least 100 intact, non-overlapping tumor nuclei were evaluated by using a Leica DMR fluorescence microscope (Leica Microsystems Nussloch GmbH, Nussloch, Germany). Samples subjected to FISH in which more than 15% of tumor nuclei had wide apart of red and green signals for more than 2 nuclei, or an absent green signal, were considered break-apart positive. Positive break-apart samples were considered to have *ALK* or *ROS1* rearrangements.

### Decision algorithm for recommendations of phase III trial design

To determine whether the RAM expression status may serve as a patient enrichment or patient stratification factor in future randomized trials of EGFR inhibitors for ABTC, the median PFS of patients who received GEMOX or C-GEMOX was further analyzed using an algorithm proposed by Freidlin B *et al.* (see supplementary information for detailed methods)^35^. Briefly, the median PFS was analyzed first to test whether the GEMOX and C-GEMOX treatment groups had the same PFS in the RAM^low^ subpopulation. If the null hypothesis was rejected, then the 2-sided 80% CI for the HR in the RAM^high^ subpopulation was analyzed to determine whether RAM expression was suitable to be used as a patient-enrichment or patient-stratification biomarker in future clinical trials. If the null hypothesis was not rejected, then the 2-sided 80% CI for the HR for all patients was analyzed to determine the value of RAM expression in future clinical trials.

### Statistical Analysis

Correlations between the results of IHC and clinicopathological factors were assessed using Fisher’s exact probability test or *t-*test. The associations between objective response rate stratified by treatment (GEMOX or C-GEMOX) and RAM expression status (RAM^high^ *vs* RAM^low^) were evaluated using the Chi-square test or Fisher’s exact test. PFS and OS were calculated using the Kaplan-Meier method and compared using the log-rank test. Univariate and multivariate Cox regression was used to estimate prognostic parameters for PFS and OS. For multivariate analysis of PFS and OS, all predictors with a *p* value <0.2 in univariate analysis were incorporated. Two-sided p-values less than 0.05 were considered statistically significant. All analysis was performed using the SAS 9.1 software package (SAS Institute, Inc., Cary, NC, USA).

## Additional Information

**How to cite this article**: Chiang, N.-J. *et al.* Expression levels of ROS1/ALK/c-MET and therapeutic efficacy of cetuximab plus chemotherapy in advanced biliary tract cancer. *Sci. Rep.* **6**, 25369; doi: 10.1038/srep25369 (2016).

## Supplementary Material

Supplementary Information

## Figures and Tables

**Table 1:** Demographic and clinical characteristic of 110 patients.

	RAM^high^ (n = 18)	RAM^low^ (n = 92)	*p*
Age, years			1.00
<60	9 (50%)	47 (51%)	
≥60	9 (50%)	45 (49%)	
Sex			0.61
Men	7 (39%)	45 (49%)	
Women	11 (61%)	47 (51%)	
ECOG PS			1.00
0	5 (28%)	29 (32%)	
1	13 (72%)	63 (69%)	
Primary site			0.02
Intra-hepatic	18 (100%)	62 (67%)	
Extra-hepatic/GB	0 (0%)	30 (33%)	
T stage			1.00
≤T_3_	14 (78%)	73 (79%)	
T_4_	4 (22%)	19 (21%)	
N stage			0.39
N_0_	3 (17%)	27 (29%)	
N_1_	15 (83%)	65 (71%)	
Disease status at entry			0.29
Locally advanced	4 (22%)	34 (37%)	
Distant metastasis	14 (78%)	58 (63%)	
Previous surgery			＜0.001
No	18 (100%)	45 (49%)	
Yes	0 (0%)	47 (51%)	
*KRAS* mutation			0.06
Negative	8 (44%)	63 (69%)	
Positive	10 (56%)	29 (32%)	
EGFR expression			0.61
Negative	6 (33%)	37 (41%)	
Positive	12 (67%)	54 (59%)	
HBsAg or anti-HCV Ab			0.56
Negative	15 (83%)	69 (75%)	
Positive	3 (17%)	23 (25%)	

RAM, ROS1/ALK/c-MET; ECOG PS, Eastern Cooperative Oncology Group performance status; GB, gallbladder; EGFR, epidermal growth factor receptor; HBsAg, surface antigen of hepatitis B virus; HCV Ab, antibody of hepatitis C virus.

**Table 2:** Therapeutic efficacies stratified by RAM expression in IHCC.

	RAM^high^ n = 18	RAM^low^ n = 62	*p*##
Objective response rate	22% (6–48)	24% (14–37)	1.000
Disease control rate#	39% (17–64)	53% (40–66)	0.423
Median PFS (months)	4.5 (1.8–8.5)	5.9 (4.2‒7.1)	0.533
Median OS (months)	5.7 (3.3–9.9)	11.7 (8.8–14.5)	0.021

RAM, ROS1/ALK/c-MET; IHCC, intrahepatic cholangiocarcinoma; PFS, progression - free survival; OS, overall survival.

^#^Disease control rate = complete response + partial response + stable disease >16 weeks.

^##^Objective response rates and disease control rates were compared using Fisher’s exact test, 2-tailed; PFS and OS were compared using log-rank tests.

**Table 3:** Therapeutic efficacies stratified by treatment arms in IHCC with RAM^low^ and RAM^high^ expression.

	C-GEMOX	GEMOX	*p*##
**RAM^low^**	**N = 28**	**N = 34**	
Disease control rate#	68% (48–84)	41% (25–59)	0.044
Median PFS (months)	7.3 (5.6–10.9)	4.9 (1.8–6.7)	0.026
Median OS (months)	14.1 (10.1–20.0)	9.6 (6.6–13.8)	0.056
**RAM^high^**	**N=10**	**N = 8**	
Disease control rate#	40% (12–74)	38% (9–76)	1.000
Median PFS (months)	4.7 (1.5–8.6)	4.5 (1.3–13.8)	0.610
Median OS (months)	6.3 (2.6–12.0)	5.2 (1.4–27.4)	0.605

RAM, ROS1/ALK/c-MET; IHCC, intrahepatic cholangiocarcinoma; C-GEMOX, cetuximab plus gemcitabine and oxaplatin; PFS, progression-free survival; OS, overall survival.

^#^Disease control rate = complete response+ partial response+ stable disease >16 weeks.

^##^Disease control rates were compared using Fisher’s exact test, 2-tailed; PFS and OS were compared using log-rank test.

**Table 4:** Univariate and multivariate Cox regression of prognostic factors for overall survival in patients with IHCC.

Variables	Univariate		Multivariate	
	Hazard ratio (95% CI)	*p*	Hazard ratio (95% CI)	*p*
Age ≥60 (*vs.* <60)	1.48 (0.92–2.36)	0.103	1.54 (0.92–2.59)	0.102
C-GEMOX (*vs.* GEMOX)	0.75 (0.48–1.18)	0.209	–	–
Male (*vs.* female)	1.62 (1.03–2.56)	0.038	2.11 (1.27–3.50)	0.004
ECOG PS 1 (*vs.* 0)	1.42 (0.87–2.34)	0.165	1.27 (0.73–2.22)	0.398
Disease status, Meta (*vs.* LA)	1.86 (1.12–3.09)	0.017	1.35 (0.76–2.41)	0.312
Prior surgery, yes (*vs.* no)	0.52 (0.32–0.85)	0.009	0.81 (0.46–1.44)	0.478
KRAS MT (*vs.* WT)	1.62 (1.01–2.59)	0.045	1.48 (0.87–2.52)	0.153
RAM^high^ (*vs.* RAM^low^)	1.86 (1.09–3.18)	0.023	2.01 (1.04–3.88)	0.039

IHCC, intrahepatic cholangiocarcinoma; C-GEMOX, cetuximab plus gemcitabine and oxaliplatin; ECOG PS, Eastern Cooperative Oncology Group performance status; Meta: metastasis; LA, locally advanced; MT: mutated-type; WT: wild-type; LA: locally advanced RAM, ROS1/ALK/c-MET.

## References

[b1] Park J. O. *et al.* Gemcitabine Plus Cisplatin for Advanced Biliary Tract Cancer: A Systematic Review. Cancer Res Treat 47, 343–361 (2015). 25989801 10.4143/crt.2014.308 PMC4509359

[b2] Okusaka T. *et al.* Gemcitabine alone or in combination with cisplatin in patients with biliary tract cancer: a comparative multicentre study in Japan. Br J Cancer 103, 469–474 (2010). 20628385 10.1038/sj.bjc.6605779 PMC2939781

[b3] Valle J. *et al.* Cisplatin plus gemcitabine versus gemcitabine for biliary tract cancer. N Engl J Med 362, 1273–1281 (2010). 20375404 10.1056/NEJMoa0908721

[b4] Chen J. S. *et al.* A KRAS mutation status-stratified randomized phase II trial of gemcitabine and oxaliplatin alone or in combination with cetuximab in advanced biliary tract cancer. Ann Oncol 26, 943–949 (2015). 25632066 10.1093/annonc/mdv035

[b5] Lee J. *et al.* Gemcitabine and oxaliplatin with or without erlotinib in advanced biliary-tract cancer: a multicentre, open-label, randomised, phase 3 study. Lancet Oncol 13, 181–188 (2012). 22192731 10.1016/S1470-2045(11)70301-1

[b6] Leone F. *et al.* A phase II, open-label, randomized clinical trial of panitumumab plus gemcitabine and oxaliplatin (GEMOX) versus GEMOX alone as first-line treatment in advanced biliary tract cancer: The Vecti-BIL study. ASCO Meeting Abstracts 33, 281 (2015).

[b7] Malka D. *et al.* Gemcitabine and oxaliplatin with or without cetuximab in advanced biliary-tract cancer (BINGO): a randomised, open-label, non-comparative phase 2 trial. Lancet Oncol 15, 819–828 (2014). 24852116 10.1016/S1470-2045(14)70212-8 PMC6372099

[b8] Van Cutsem E. *et al.* Cetuximab plus irinotecan, fluorouracil, and leucovorin as first-line treatment for metastatic colorectal cancer: updated analysis of overall survival according to tumor KRAS and BRAF mutation status. J Clin Oncol 29, 2011–2019 (2011). 21502544 10.1200/JCO.2010.33.5091

[b9] Pirker R. *et al.* EGFR expression as a predictor of survival for first-line chemotherapy plus cetuximab in patients with advanced non-small-cell lung cancer: analysis of data from the phase 3 FLEX study. Lancet Oncol 13, 33–42 (2012). 22056021 10.1016/S1470-2045(11)70318-7

[b10] Cappuzzo F. *et al.* MET increased gene copy number and primary resistance to gefitinib therapy in non-small-cell lung cancer patients. Ann Oncol 20, 298–304 (2009). 18836087 10.1093/annonc/mdn635 PMC2733067

[b11] Miyamoto M. *et al.* Prognostic significance of overexpression of c-Met oncoprotein in cholangiocarcinoma. Br J Cancer 105, 131–138 (2011). 21673683 10.1038/bjc.2011.199 PMC3137414

[b12] Bergethon K. *et al.* ROS1 rearrangements define a unique molecular class of lung cancers. J Clin Oncol 30, 863–870 (2012). 22215748 10.1200/JCO.2011.35.6345 PMC3295572

[b13] Kwak E. L. *et al.* Anaplastic lymphoma kinase inhibition in non-small-cell lung cancer. N Engl J Med 363, 1693–1703 (2010). 20979469 10.1056/NEJMoa1006448 PMC3014291

[b14] Gu T. L. *et al.* Survey of tyrosine kinase signaling reveals ROS kinase fusions in human cholangiocarcinoma. Plos One 6, e15640 (2011). 21253578 10.1371/journal.pone.0015640 PMC3017127

[b15] Saborowski A. *et al.* Mouse model of intrahepatic cholangiocarcinoma validates FIG-ROS as a potent fusion oncogene and therapeutic target. Proc Natl Acad Sci USA 110, 19513–19518 (2013). 24154728 10.1073/pnas.1311707110 PMC3845141

[b16] Shaw A. T. *et al.* Ceritinib in ALK-rearranged non-small-cell lung cancer. N Engl J Med 370, 1189–1197 (2014). 24670165 10.1056/NEJMoa1311107 PMC4079055

[b17] Shaw A. T. *et al.* Crizotinib in ROS1-rearranged non-small-cell lung cancer. N Engl J Med. 371, 1963–1971 (2014). 25264305 10.1056/NEJMoa1406766 PMC4264527

[b18] Chan-On W. *et al.* Exome sequencing identifies distinct mutational patterns in liver fluke-related and non-infection-related bile duct cancers. Nat Genet 45, 1474–1478 (2013). 24185513 10.1038/ng.2806

[b19] Chiu J. W.-Y. *et al.* Next-generation sequencing: Profiling gallbladder cancer (GBC). ASCO Meeting Abstracts 33, 286 (2015).

[b20] Holcombe R. F. *et al.* Tumor profiling of biliary tract carcinomas to reveal distinct molecular alterations and potential therapeutic targets. ASCO Meeting Abstracts 33, 285 (2015).

[b21] Ross J. S. *et al.* Comprehensive genomic profiling of biliary tract cancers to reveal tumor-specific differences and genomic alterations. ASCO Meeting Abstracts 33, 231 (2015).

[b22] Andersen J. B. *et al.* Genomic and genetic characterization of cholangiocarcinoma identifies therapeutic targets for tyrosine kinase inhibitors. Gastroenterology 142, 1021–1031 e1015 (2012). 22178589 10.1053/j.gastro.2011.12.005 PMC3413201

[b23] Sia D. *et al.* Integrative molecular analysis of intrahepatic cholangiocarcinoma reveals 2 classes that have different outcomes. Gastroenterology 144, 829–840 (2013). 23295441 10.1053/j.gastro.2013.01.001 PMC3624083

[b24] Aljohani H. *et al.* ROS1 amplification mediates resistance to gefitinib in glioblastoma cells. Oncotarget 6, 20388–20395 (2015). 25978031 10.18632/oncotarget.3981 PMC4653012

[b25] Davies K. D. *et al.* Resistance to ROS1 inhibition mediated by EGFR pathway activation in non-small cell lung cancer. Plos One 8, e82236 (2013). 24349229 10.1371/journal.pone.0082236 PMC3862576

[b26] Dempke W. C. & Heinemann V. Resistance to EGF-R (erbB-1) and VEGF-R modulating agents. Eur J Cancer 45, 1117–1128 (2009). 19124237 10.1016/j.ejca.2008.11.038

[b27] Miyanaga A. *et al.* Activity of EGFR-tyrosine kinase and ALK inhibitors for EML4-ALK-rearranged non-small-cell lung cancer harbored coexisting EGFR mutation. BMC Cancer 13, 262 (2013). 23714228 10.1186/1471-2407-13-262 PMC3671182

[b28] Shaw A. T. *et al.* Clinical features and outcome of patients with non-small-cell lung cancer who harbor EML4-ALK. J Clin Oncol 27, 4247–4253 (2009). 19667264 10.1200/JCO.2009.22.6993 PMC2744268

[b29] Yang J. J. *et al.* Lung cancers with concomitant EGFR mutations and ALK rearrangements: diverse responses to EGFR-TKI and crizotinib in relation to diverse receptors phosphorylation. Clin Cancer Res 20, 1383–1392 (2014). 24443522 10.1158/1078-0432.CCR-13-0699

[b30] Le X. *et al.* Detection of Crizotinib-Sensitive Lung Adenocarcinomas With MET, ALK, and ROS1 Genomic Alterations via Comprehensive Genomic Profiling. Clin Lung Cancer 16, e105–109 (2015). 25922291 10.1016/j.cllc.2015.03.002 PMC4418215

[b31] Raedler L. A. Zykadia (Ceritinib) Approved for Patients with Crizotinib-Resistant ALK -Positive Non-Small-Cell Lung Cancer. Am Health Drug Benefits 8, 163–166 (2015). 26629283 PMC4665050

[b32] Wu Y. C. *et al.* Comparison of IHC, FISH and RT-PCR methods for detection of ALK rearrangements in 312 non-small cell lung cancer patients in Taiwan. Plos One 8, e70839 (2013). 23951022 10.1371/journal.pone.0070839 PMC3737393

[b33] Lee K. H. *et al.* Clinical and pathological significance of ROS1 expression in intrahepatic cholangiocarcinoma. BMC Cancer 15, 721 (2015). 26475437 10.1186/s12885-015-1737-4 PMC4609147

[b34] Lee H. J. *et al.* ROS1 receptor tyrosine kinase, a druggable target, is frequently overexpressed in non-small cell lung carcinomas via genetic and epigenetic mechanisms. Ann Surg Oncol. 20, 200–208 (2013). 22915320 10.1245/s10434-012-2553-6

[b35] Freidlin B., McShane L. M., Polley M. Y. & Korn E. L. Randomized phase II trial designs with biomarkers. J Clin Oncol 30, 3304–3309 (2012). 22869885 10.1200/JCO.2012.43.3946 PMC3434989

