# Supplementary material for: Expression levels of ROS1/ALK/c-MET and therapeutic efficacy of cetuximab plus chemotherapy in advanced biliary tract cancer
Source: Sci Rep. 2016 May 3;6:25369. doi: 10.1038/srep25369 (PMC4853728; doi:10.1038/srep25369)
Supplement: Supplementary Information [file srep25369-s1.doc]

**Expression levels of ROS1/ALK/c-MET and therapeutic efficacy of cetuximab plus chemotherapy in advanced biliary tract cancer**

Nai-Jung Chiang^1,2,3*^, Chiun Hsu^4,5*^, Jen‐Shi Chen^6＊^, Hsiao-Hui Tsou^7,8^, Ying-Ying Shen^9^, Yee Chao^10^, Ming-Huang Chen^10^, Ta-Sen Yeh^11^, Yan-Shen Shan^1,12**^, Shiu-Feng Huang^9**^, Li‐Tzong Chen^2,3,13,14**^

^1^Institute of Clinical Medicine, College of Medicine, National Cheng Kung University, Tainan, Taiwan.

^2^National Institute of Cancer Research, National Health Research Institutes, Tainan, Taiwan.

^3^Division of Hematology and Oncology, Department of Internal Medicine, National Cheng Kung University Hospital, Tainan, Taiwan.

^4^Department of Oncology, National Taiwan University Hospital, and National Taiwan University Cancer Center, Taipei, Taiwan.

^5^Graduate Institute of Oncology, National Taiwan University College of Medicine, Taipei, Taiwan.

^6^Division of Hematology and Oncology, Department of Internal Medicine, Linkou Chang Gung Memorial Hospital and Chang Gung University, Taoyuan, Taiwan.

^7^Division of Biostatistics and Bioinformatics, Institute of Population Health Sciences, National Health Research Institutes, Miaoli, Taiwan.

^8^Graduate Institute of Biostatistics, College of Public Health, China Medical University, Taichung, Taiwan.

^9^Institute of Molecular and Genomic Medicine, National Health Research Institutes, Miaoli, Taiwan.

^10^Department of Oncology, Taipei Veterans General Hospital, Taipei, Taiwan.

^11^Department of Surgery, Linkou Chang Gung Memorial Hospital and Chang Gung University, Taoyuan, Taiwan.

^12^Department of Surgery, National Cheng Kung University Hospital, Tainan, Taiwan.

^13^Institute of Clinical Pharmacology, College of Medicine, National Cheng Kung University, Tainan, Taiwan.

^14^Department of Internal Medicine, Kaohsiung Medical University Hospital, Kaohsiung Medical University, Kaohsiung, Taiwan.

^*^Co-first authors

^**^Equal contribution

Correspondence and requests for materials should be addressed to Y.S.S, S.F.H, and L.T.C. (email: [ysshan@mail.ncku.edu.tw](mailto:ysshan@mail.ncku.edu.tw) or [sfhuang@nhri.org.tw](mailto:sfhuang@nhri.org.tw) or [leochen@nhri.org.tw](mailto:leochen@nhri.org.tw))

**Supplementary table 1.**

**Distribution of RAM expression in biliary tract cancer**

|  | All sites, n (%) | | EHCC/GB, n (%) | | IHCC, n (%) | | *P* |
| --- | --- | --- | --- | --- | --- | --- | --- |
| **c-MET** | | | | | | | |
|  | 121 |  | 33 |  | 88 |  |  |
| 0 | 29 | ( 24%) | 5 | ( 15%) | 24 | ( 27%) | 0.20 |
| 1+ | 39 | ( 32%) | 13 | ( 40%) | 26 | ( 30%) |  |
| 2+ | 47 | ( 39%) | 15 | ( 46%) | 32 | ( 37%) |  |
| 3+ | 6 | ( 5%) | 0 | ( 0%) | 6 | ( 7%) |  |
| **ALK** | | | | | | | |
|  | 110 |  | 30 |  | 80 |  |  |
| 0 | 31 | ( 28%) | 6 | ( 20%) | 25 | ( 31%) | 0.26 |
| 1+ | 36 | ( 33%) | 13 | ( 43%) | 23 | ( 29%) |  |
| 2+ | 38 | ( 35%) | 11 | ( 37%) | 27 | ( 34%) |  |
| 3+ | 5 | ( 5%) | 0 | ( 0%) | 5 | ( 6%) |  |
| **ROS1** | | | | | | | |
|  | 117 |  | 32 |  | 85 |  |  |
| 0 | 35 | ( 30%) | 11 | ( 34%) | 24 | ( 28%) | 0.22 |
| 1+ | 37 | ( 32%) | 12 | ( 38%) | 25 | ( 30%) |  |
| 2+ | 36 | ( 31%) | 9 | ( 28%) | 27 | ( 32%) |  |
| 3+ | 9 | ( 8%) | 0 | ( 0%) | 9 | ( 11%) |  |

Online only

RAM, ROS1/ALK/c-MET; EHCC, extrahepatic cholangiocarcinoma; GB. gallbladder; IHCC, intrahepatic cholangiocarcinoma.

**Supplementary table 2.**

**Demographic and clinical characteristic of patients with RAM^high^ vs RAM^low^ IHCC**

|  | RAM^high^  (n=18) | RAM^low^  (n=62) | *p* |
| --- | --- | --- | --- |
| **Age, years** | | | 0.90 |
| Median (range) | 61 (41-77) | 60 (32-80) |  |
| **Sex** | | | 0.43 |
| Men | 7 (39%) | 32 (52%) |  |
| Women | 11 (61%) | 30 (48%) |  |
| **ECOG PS** | | | 1.00 |
| 0 | 5 (28%) | 18 (29%) |  |
| 1 | 13 (72%) | 44 (71%) |  |
| **T stage** | | | 1.00 |
| **≤** T_3_ | 14 (78%) | 49 (79%) |  |
| T_4_ | 4 (22%) | 13 (21%) |  |
| **N stage** | | | 0.37 |
| N_0_ | 3 (17%) | 18 (29%) |  |
| N_1_ | 15 (83%) | 44 (71%) |  |
| **Disease status at entry** | | | 0.77 |
| Locally advanced | 4 (22%) | 18 (29%) |  |
| Distant metastasis | 14 (78%) | 44 (71%) |  |
| **Previous surgery** | | | <0.01 |
| No | 18 (100%) | 36 (58%) |  |
| Yes | 0 (0%) | 26 (42%) |  |
| ***KRAS* status** |  | | 0.09 |
| Negative | 8 (44%) | 43 (69%) |  |
| Positive | 10 (56%) | 19 (31%) |  |
| **EGFR expression** | | | 0.59 |
| Negative | 6 (33%) | 27 (44%) |  |
| Positive | 12 (67%) | 35 (57%) |  |
| **HBsAg or anti-HCV Ab** | | | 0.25 |
| Negative | 15 (83%) | 42 (68%) |  |
| Positive | 3 (17%) | 20 (32%) |  |

Online only

RAM, ROS1/ALK/c-MET; ECOG PS, eastern cooperative oncology group performance status; GB, gallbladder; EGFR, epidermal growth factor receptor; HBsAg, surface antigen of hepatitis B virus; HCV Ab, antibody of hepatitis C virus.

**Supplementary table 3.**

**Therapeutic efficacies stratified by treatment in 110 patients with RAM assessment**

|  | **C-GEMOX**  n=54 | **GEMOX**  n=56 | *p*^＃＃^ |
| --- | --- | --- | --- |
| Objective response rate | 28% (17-42) | 14% (6-26) | 0.103 |
| Disease control rate^＃^ | 57% (43-71) | 40% (27-53) | 0.085 |
| PFS (months) | 6.7 (4.2-8.1) | 4.5 (2.3-6.0) | 0.105 |
| OS (months) | 11.2 (8.8-13.9) | 9.6 (6.6-12.4) | 0.430 |
| RAM, ROS1/ALK/c-MET; IHCC, intrahepatic cholangiocarcinoma; PFS. Progression=free survival; OS, overall survival.  ^＃^Disease control rate = complete response + partial response + stable disease > 16 weeks  ^＃＃^Objective response rates and disease control rates were compared using Fisher’s exact test, 2-tailed; PFS and OS were compared using log-rank tests. | | | |

**Supplementary table 4.**

**Therapeutic efficacies stratified by treatment arms in IHCC with *KRAS* wild-type or mutation**

|  |  | C-GEMOX | GEMOX |  |
| --- | --- | --- | --- | --- |
| ***KRAS* wild-type** |  | N=19 | N=24 | *p*^＃＃^ |
| Objective response rate |  | 47% (24-71) | 13% (3-32) | 0.017 |
| Disease control rate^＃^ |  | 74% (49-91) | 54% (33-74) | 0.221 |
| Median PFS (months) |  | 7.4 (5.6-12.0) | 5.9 (2.3-7.5) | 0.032 |
| Median OS (months) |  | 15.3 (10.1-23.0) | 12.3 (7.5-16.2) | 0.210 |
| ***KRAS* mutation** |  | N=10 | N=8 | *p*^＃＃^ |
| Objective response rate |  | 22% (3-60) | 10% (0-45) | 0.582 |
| Disease control rate^＃^ |  | 56% (21-86) | 10% (0-45) | 0.057 |
| Median PFS (months) |  | 7.0 (1.5-16.2) | 1.9 (1.1-5.0) | 0.339 |
| Median OS (months) |  | 10.9 (2.4-24.8) | 6.7 (3.7-7.2) | 0.066 |
| Online only | | | |  |

RAM, ROS1/ALK/c-MET; IHCC, intrahepatic cholangiocarcinoma; C-GEMOX, cetuximab plus gemcitabine and oxaplatin; PFS. Progression=free survival; OS, overall survival.

^＃^Disease control rate=complete response + partial response + stable disease > 16 weeks

^＃＃^Disease control rates were compared using Fisher’s exact test, 2-tailed; PFS and OS were compared using log-rank test.

**Supplementary table 5.**

**Univariate and multivariate analyses of prognostic factors for progression-free survival in 80 patients of IHCC**

| **Variables** | Univariate | |  | Multivariate^＃^ | |
| --- | --- | --- | --- | --- | --- |
|  | Hazard ratio (95% CI) | *p* |  | Hazard ratio (95% CI) | *p* |
| Age ≥ 60 (*vs* < 60) | 1.19 (0.76,1.85) | 0.455 |  | - | - |
| C-GEMOX (*vs* GEMOX) | 0.69 (0.44,1.08) | 0.102 |  | 0.66 (0.42 - 1.04) | 0.072 |
| Male (*vs* female) | 1.50 (0.95,2.36) | 0.077 |  | 1.56 (0.99 – 2.46) | 0.055 |
| ECOG PS 1 (*vs* 0) | 1.13 (0.70,1.85) | 0.612 |  | - | - |
| Disease status, Meta (*vs* LA) | 1.29 (0.79,2.13) | 0.311 |  | - | - |
| Prior surgery, yes (*vs* no) | 0.80 (0.50,1.29) | 0.362 |  | - | - |
| KRAS MT (*vs* WT) | 1.24 (0.78,1.97) | 0.360 |  | - | - |
| RAM^high^ (*vs* RAM^low^) | 1.18 (0.70,2.01) | 0.536 |  | - | - |

Online only

IHCC, intrahepatic cholangiocarcinoma; C-GEMOX, cetuximab plus gemcitabine and oxaliplatin; ECOG PS, eastern cooperative oncology group performance status; Meta: metastasis; LA, locally advanced; MT: mutated-type; WT: wild-type; LA: locally advanced; RAM, ROS1/ALK/c-MET.

^＃^All predictors with *p* < 0.2 in the univariate analysis were included.

**Supplementary table 6**

**Decision algorithm for recommendation of phase III trial design in IHCC patients**

| Biomarker |  | RAM^low^ (Step 1,HR_(+)_ one-sided α=0.1) | | | |  | RAM^high^ (Step 2B, 80%CI for HR(-)) | | | |  | Comment |
| --- | --- | --- | --- | --- | --- | --- | --- | --- | --- | --- | --- | --- |
|  |  | C-GEMOX | GEMOX |  |  |  | C-GEMOX | GEMOX |  |  |  |  |

| Positive prevalence (RAM^low^, 77.5%) |  | mPFS | mPFS | HR | *p* |  | mPFS | mPFS | HR | 80%CI |  |  |
| --- | --- | --- | --- | --- | --- | --- | --- | --- | --- | --- | --- | --- |
|  |  | 7.3 | 4.9 | 1.78 | 0.028 |  | 4.7 | 4.5 | 0.77 | (0.39,1.50) |  | Phase III trial: biomarker-stratified design |

Online only

HR, hazard ratio; CI, confidence interval; mPFS, median progression-free survival (months); RAM, ROS1/ALK/c-MET

**Decision algorithm for recommendations of phase III trial design**

The analysis algorithm was adopted from Fredlin B et al[^1^](#_ENREF_1). In step 1 the efficacy in biomarker-positive (RAM^low^) subgroups was analyzed. If C-GEMOX showed superior efficacy in step 1 (null hypothesis rejected), then in step 2B the efficacy in biomarker-negative (RAM^high^) subgroups was analyzed to see whether the biomarkers (RAM expression level) is useful for patient enrichment (CI_HR_ below 1.3), for patient stratification (CI_HR_ includes 1.3 or 1.5). If CI_HR_ was above 1.5, which meant that C-GEMOX was also efficacious in biomarker-negative subgroups, future phase III trials may be done without the use of the biomarkers.


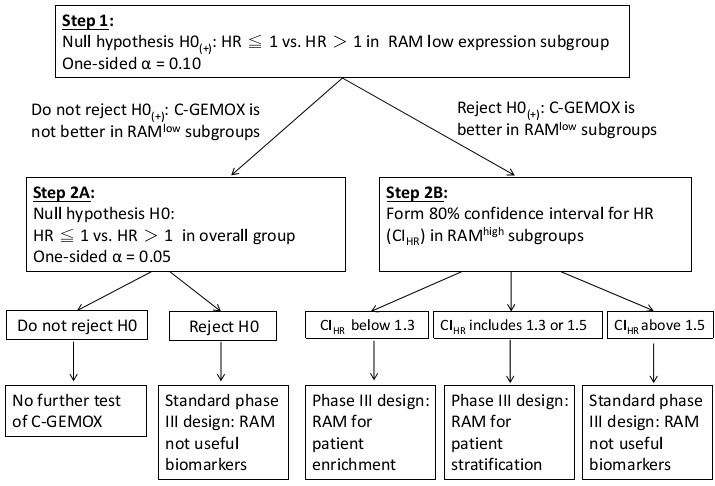

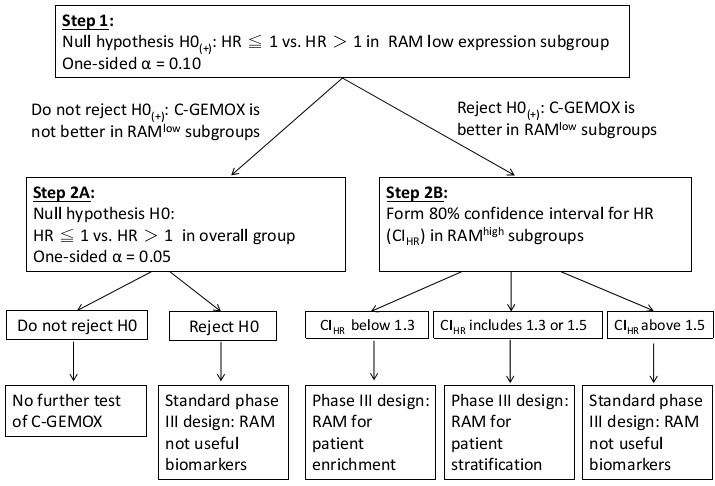
If C-GEMOX did not show superior efficacy in step 1 (null hypothesis not rejected), the biomarkers were considered not useful either for patient enrichment or patient stratification. Further phase III trials of C-GEMOX may be designed if efficacy was shown in the overall population (step 2A).

1. Freidlin, B., McShane, L. M., Polley, M. Y. & Korn, E. L. Randomized phase II trial designs with biomarkers*. J Clin Onc*o**l** 30, 3304-3309 (2012).
